# Prevalence of Periodontitis and Tooth Loss Among Older Adult Women With Low Bone Mineral Density: Protocol for a Cross-Sectional Study

**DOI:** 10.2196/96991

**Published:** 2026-08-28

**Authors:** Shirmila Syamala, Sukumaran Anil, Jafar Alabdallah, Seham Alyafei, Yasmine Eid, Aishah Althuwaini, Yasemin Sezgin

**Affiliations:** 1 Department of Geriatrics and Long-Term Care, Hamad Medical Corporation, P.O. Box 3050, Al Rumailah Street, Doha, Al Sadd, Qatar, 974 44397855; 2 Center of Excellence in Precision Medicine and Digital Health, Department of Physiology, Faculty of Dentistry, Chulalongkorn University, Bangkok, Thailand; 3 University of Doha for Science and Technology, Doha, Baladīyat ad Dawḩah, Qatar; 4 Hamad Dental Center, Hamad Medical Corporation, Doha, Qatar; 5 Department of Periodontology, Faculty of Dentistry, Baskent University, Ankara, Turkey

**Keywords:** periodontitis, bone mineral density, osteoporosis, tooth loss, matrix metalloproteinase-8, point-of-care testing, older women, geriatric oral health

## Abstract

**Background:**

Periodontitis and low bone mineral density (BMD) are both highly prevalent among older women and share inflammatory and metabolic pathways, yet their co-occurrence and combined impact on tooth loss remain insufficiently characterized, particularly in Middle Eastern populations. Active matrix metalloproteinase-8 (aMMP-8), measured by point-of-care testing (PoCT) in oral rinse, is a candidate biomarker that may support periodontal screening in geriatric settings.

**Objective:**

The primary objective is to determine and compare the prevalence and severity of periodontitis, classified using the 2017 American Academy of Periodontology and European Federation of Periodontology (AAP and EFP) classification system, between older women with low BMD (osteopenia or osteoporosis) and age-matched women with normal BMD. Secondary objectives are to compare clinical periodontal parameters and tooth loss between groups; to evaluate the diagnostic performance of aMMP-8 PoCT against the clinical periodontal diagnosis; and to examine associations among BMD, periodontal status, aMMP-8, and risk factors.

**Methods:**

This single-center, cross-sectional study will recruit postmenopausal women aged 50 years or older who attend the geriatric clinics at Rumailah Hospital, Hamad Medical Corporation, Doha, Qatar. Cases are women with a dual-energy X-ray absorptiometry (DXA) T-score of −1.0 or lower; controls are women with a T-score greater than −1.0. Each case will be individually matched to a control by age within 3 years. All participants will undergo a full-mouth periodontal examination by a single calibrated examiner, assessment of tooth loss, aMMP-8 oral rinse PoCT (20 ng/mL cutoff), and comprehensive geriatric assessment and complete a structured questionnaire, with DXA results obtained from records. The target sample is 400 participants (200 per group). The primary matched comparison of periodontitis prevalence between groups will use the McNemar tests and conditional logistic regression; secondary analyses will use chi-square, *t*, or Mann-Whitney *U* tests; correlation; and regression in SPSS (version 27; 2-sided α of .05).

**Results:**

This protocol describes a study in its data collection phase. The study received ethics approval on September 1, 2025 (MRC-01-25-782), and it is funded by the Hamad Medical Corporation Medical Research Center (funding awarded in January 2026). Screening of eligible participants who met the inclusion criteria started in June 2026. Recruitment is anticipated to commence in October 2026 and is expected to be completed by October 2027. The primary results are anticipated for publication in early 2028. No outcome data are available at the time of submission.

**Conclusions:**

The study will generate local evidence on the relationship between skeletal and oral health in older women and will evaluate whether aMMP-8 PoCT can serve as a practical, noninvasive screening adjunct in geriatric clinics. The findings are intended to inform integrated preventive and referral pathways in Qatar. Given the cross-sectional design, the study will characterize associations rather than establish causality.

## Introduction

### Background

Periodontitis is a chronic inflammatory disease of the tooth-supporting tissues, characterized by progressive destruction of the periodontal ligament and alveolar bone, which can lead to tooth loss if left untreated. It is a major public health concern worldwide: the Global Burden of Disease 2021 study estimated that more than 1 billion people are affected by severe periodontitis, with an age-standardized prevalence of approximately 12.5%, and projected that both severe periodontitis and edentulism will increase substantially by 2050 [1]. Additionally, updated Global Burden of Disease modeling projects that the burden of periodontal disease will continue to rise through 2035 [2]. The prevalence and severity of periodontitis increase with age, reflecting the cumulative effect of risk factors over time, as well as age-related changes in immune function and the oral microbiome [3].

Low bone mineral density (BMD) is also common among older people, particularly postmenopausal women; population studies estimate a high prevalence of osteopenia and osteoporosis among postmenopausal women, including in Middle Eastern populations [4]. Osteoporosis is a systemic skeletal disorder characterized by reduced bone mass and microarchitectural deterioration of bone tissue, which increases fracture risk and adversely affects quality of life [5,6]. The relationship between periodontitis and low BMD has been examined in numerous studies, and current evidence supports an association and shared pathophysiological mechanisms, including chronic inflammation and dysregulated bone metabolism [5-7]. Estrogen deficiency after menopause promotes osteoclast-mediated resorption of both systemic and alveolar bone, providing a plausible biological link between the 2 conditions [8]. A systematic review and meta-analysis reported that postmenopausal women with osteoporosis have significantly greater clinical attachment loss, probing depth, gingival recession, and bleeding on probing than women without osteoporosis, consistent with the hypothesis that reduced systemic bone density may accompany a heavier periodontal burden [9]. Clinical and radiographic studies in postmenopausal women report comparable associations [10].

Active matrix metalloproteinase-8 (aMMP-8), also known as neutrophil collagenase or collagenase-2, has emerged as a supporting biomarker for periodontal tissue breakdown. It is the principal collagenase associated with active periodontal destruction, and chairside point-of-care testing (PoCT) lateral-flow immunoassays that measure aMMP-8 in mouth rinse have been developed as rapid, noninvasive adjuncts to clinical examination [11,12]. Studies using a 20 ng/mL cutoff have shown that aMMP-8 can help distinguish periodontal health from disease and correlate with clinical periodontal parameters, although diagnostic performance varies across populations and with coexisting inflammatory conditions [12,13]. A recent systematic review and meta-analysis found that the salivary aMMP-8 point-of-care test has moderate pooled diagnostic accuracy for periodontitis in adults [14]. Because oral rinse collection is simple and well tolerated, aMMP-8 PoCT is attractive for screening in settings where a full periodontal examination is not routinely feasible, such as geriatric clinics.

Despite this growing body of evidence, comprehensive studies that jointly investigate the prevalence of periodontitis, tooth loss, and low BMD in older adults remain limited, especially in the Middle East. The present study addresses this gap by determining the prevalence and severity of periodontitis and tooth loss among older women with low BMD compared with age-matched women with normal BMD, while incorporating aMMP-8 PoCT as a supporting diagnostic tool and examining associated risk factors such as age, smoking status, and medical history, each of which is independently associated with periodontal breakdown [15,16].

Qatar is undergoing a rapid demographic transition, with a growing older population and an increasing number of postmenopausal women who require specialized care [17]. This study aligns with national health priorities that emphasize integrated, evidence-based care for the aging population [18]. By focusing on older postmenopausal women, a growing and comparatively understudied group locally, the study will provide data to inform local clinical guidance, public health strategy, and health care planning that bridges bone health and oral health.

### Objectives

The primary objective is to determine and compare the prevalence and severity of periodontitis, classified using the 2017 American Academy of Periodontology and European Federation of Periodontology (AAP and EFP) classification system [19,20], among older women with low BMD (osteopenia or osteoporosis) and age-matched women with normal BMD. The primary end point is the presence of periodontitis (a case defined by interdental clinical attachment loss detectable at 2 or more nonadjacent teeth, or buccal or oral attachment loss of at least 3 mm with pocketing greater than 3 mm at 2 or more teeth, per the 2017 case definition), ascertained at the single study visit. The primary statistical comparison is the difference in periodontitis prevalence between the low-BMD and normal-BMD groups, evaluated across the 1:1 age-matched pairs with the McNemar test. Periodontitis severity, expressed as the ordinal stage, is a coprimary descriptor analyzed as a matched secondary comparison.

The secondary objectives are to compare mean clinical periodontal parameters (probing depth, clinical attachment level, and percentage of sites with bleeding on probing) between groups; to determine and compare the prevalence of tooth loss and the reported reasons for tooth loss; to determine the prevalence of aMMP-8 positivity (20 ng/mL or higher) in oral rinse and to evaluate its diagnostic performance against the clinical periodontal diagnosis; to assess the correlation between aMMP-8 levels and clinical periodontal parameters; to examine the relationship between the severity of low BMD (by T-score category) and the severity of periodontitis; and to explore associations among aMMP-8 levels, BMD, and demographic, medical, and lifestyle risk factors. A final objective is to evaluate the potential of aMMP-8 PoCT as a noninvasive supporting tool for periodontal screening among women attending geriatric clinics.

## Methods

### Study Design

This study will use a cross-sectional design to investigate the prevalence of and associations among periodontitis, tooth loss, aMMP-8 levels in oral rinse, and low BMD in women aged 50 years or older. Clinical periodontal examination and biochemical biomarker analysis will be performed at a single study visit. The protocol was developed in accordance with the STROBE (Strengthening the Reporting of Observational Studies in Epidemiology) statement [21], with consideration of relevant items from the SPIRIT (Standard Protocol Items: Recommendations for Interventional Trials) statement to promote completeness and transparency [22]. The study will be conducted by a multidisciplinary team of geriatricians, dental clinicians, and trained research assistants; a single calibrated dental examiner will perform all periodontal examinations to ensure consistency. All team members are trained in Good Clinical Practice, and study procedures will be overseen by the principal investigator at Hamad Medical Corporation (HMC).

### Study Setting and Participants

The study population comprises postmenopausal women aged 50 years or older who reside in Qatar, regardless of nationality, and who attend the geriatric clinics of Rumailah Hospital, HMC. This includes Qatari nationals and long-term residents from diverse backgrounds, reflecting the diversity of the clinic population. Participants will be recruited into 2 groups on the basis of existing dual-energy X-ray absorptiometry (DXA) scans: cases are women with low BMD (osteopenia or osteoporosis), and controls are women with normal BMD. Eligibility criteria are summarized in Textbox 1.

For BMD classification, the World Health Organization criteria will be applied to the lowest T-score at the lumbar spine (L1-L4), femoral neck, or total hip [23]: normal BMD is a T-score greater than −1.0, osteopenia is a T-score between −1.0 and −2.5, and osteoporosis is a T-score of −2.5 or lower. Accordingly, cases are defined as women with a T-score of −1.0 or lower (ie, osteopenia or osteoporosis), and controls as women with a T-score greater than −1.0. Women who meet a clinical diagnosis of osteoporosis on grounds other than DXA will also be eligible as cases; for a participant without an eligible DXA T-score, a high fracture-risk score is defined a priori as a documented prior fragility fracture or a fracture risk assessment tool–based 10-year fracture probability at or above the intervention threshold, operationalized as a major osteoporotic fracture probability of 20% or higher or a hip fracture probability of 3% or higher [24].

Textbox 1. **Inclusion and exclusion criteria.**

Inclusion criteria

- Women aged 50 years or older attending geriatric clinics at Hamad Medical Corporation
- Residents of Qatar
- Postmenopausal status, defined as absence of menstruation for at least 12 consecutive months not attributable to another medical cause
- A dual-energy X-ray absorptiometry (DXA) result within the preceding 2 years confirming bone mineral density status
- Cases: T-score of −1.0 or lower at the lumbar spine, femoral neck, or total hip or a clinical diagnosis of osteoporosis on other grounds (fragility fracture or high fracture-risk score)
- Controls: T-score greater than −1.0 at all measured sites
- For a participant without an eligible DXA T-score, a high fracture-risk score is defined as a documented prior fragility fracture or a fracture risk assessment tool–based 10-year fracture probability at or above the intervention threshold (major osteoporotic fracture probability of 20% or higher or hip fracture probability of 3% or higher)
- Able to provide an adequate oral rinse sample for active matrix metalloproteinase-8 (aMMP-8) testing
- Able and willing to provide written informed consent

Exclusion criteria

- Systemic disease materially affecting bone metabolism or periodontal health, such as uncontrolled diabetes mellitus, rheumatoid arthritis, Paget disease, or other metabolic bone disease (other than osteoporosis or osteopenia)
- Current or recent (within 6 months) systemic antibiotic therapy for periodontal reasons
- Nonsurgical or surgical periodontal treatment within the preceding 6 months
- Use of medications associated with gingival overgrowth (eg, cyclosporine A, phenytoin, or nifedipine)
- Fewer than 8 natural teeth present, excluding third molars
- Cognitive impairment that would preclude informed consent or participation
- Food or fluid intake within 1 hour before oral rinse collection

### Matching of Cases and Controls

Controls will be individually matched to cases by age. For each recruited case, 1 control with normal BMD will be selected from the same clinic population, with an age within 3 years of the case, to minimize selection bias related to the source population. Individual (rather than frequency) matching was chosen to improve comparability by age, a strong shared determinant of both periodontal disease and BMD. Where more than 1 eligible control is available for a given case, the closest match by age will be selected. Matching variables will be recorded to allow the matched structure to be accounted for in the analysis.

### Sample Size

The sample size was calculated to provide adequate power to detect a difference in the prevalence of periodontitis between women with low BMD and age-matched controls with normal BMD. On the basis of the literature, the prevalence of periodontitis in older women without osteoporosis was estimated at 40% (p1=0.40), and a clinically meaningful minimum difference of 15% was assumed, giving an expected prevalence of 55% (p2=0.55) in the low-BMD group [7,9]. Using the formula for comparing 2 independent proportions, n = [p1(1 – p1) + p2(1 – p2)] × (Z_alpha/2 + Z_beta)^2^ / (p1 – p2)^2^, with Z_alpha/2=1.96 (95% confidence) and Z_beta=0.84 (80% power) and a 1:1 case-to-control ratio, the estimated minimum requirement is approximately 170 participants per group, or 340 participants in total.

To account for nonresponse, incomplete data, incomplete DXA records, and participant withdrawal and to preserve balanced group sizes after matching, the recruitment target was set at 200 participants per group, for a total of 400 participants. This corresponds to an allowance of approximately 18% above the statistical minimum of 340, rather than a 10% allowance; the larger allowance was adopted deliberately because complete data across DXA records, full-mouth periodontal examination, aMMP-8 testing, and comprehensive geriatric assessment are required for each participant and because rounding to 200 per group simplifies balanced enrollment of matched pairs. Approximately 600 women will be screened to achieve this target. The target of 400 also provides adequate power to detect the anticipated between-group differences in aMMP-8 levels, based on reported effect sizes of 0.5 to 0.8 between periodontally healthy and diseased groups, and is consistent with the reported prevalence of low BMD among postmenopausal women in Qatar [17].

### Data Collection

Data will be collected at a single study visit through a structured questionnaire, medical record review, clinical oral examination, aMMP-8 PoCT, and review of existing radiographs. A trained researcher will administer the questionnaire to collect demographic information (age, education level, and socioeconomic status where feasible), medical history (systemic diseases, current medications, history of hormone replacement therapy, and smoking status categorized as never, past, or current), the SARC-F screen for sarcopenia [25], and the self-reported reason for any tooth loss. Medical record review will confirm BMD status and DXA T-scores for the lumbar spine and femoral neck, relevant medical history, medication use, and details of the comprehensive geriatric assessment [26], including functional status, frailty (Clinical Frailty Scale [27]), and sarcopenia, subject to participant consent and ethical approval.

The clinical oral examination will be performed by a single calibrated examiner, who will record periodontal and dental parameters as described below. Existing panoramic radiographs and DXA reports will be reviewed where available. As an additional measure of oral function relevant to geriatric assessment, articulatory oral motor skill will be assessed using oral diadochokinesis: participants will be asked to repeat the syllable “ta” as rapidly as possible for 5 seconds, and a rate below 6.0 repetitions per second will be classified as low articulatory oral motor skill [28].

### Periodontal Examination

Periodontal disease will be diagnosed and classified using the 2017 World Workshop classification (staging and grading) based on probing depth, clinical attachment loss, and bleeding on probing [19]. A single trained and calibrated examiner will perform a full-mouth examination of all present teeth except third molars, at 6 sites per tooth (mesiobuccal, buccal, distobuccal, mesiolingual, lingual, and distolingual). Probing depth, gingival recession, and clinical attachment loss will be recorded to the nearest whole millimeter using a calibrated UNC-15 probe. Clinical attachment loss will be calculated for each site as probing depth plus gingival recession where recession is present, or probing depth minus the distance from the cementoenamel junction where gingival enlargement is present. Bleeding on probing will be recorded dichotomously within 10 seconds of probing and expressed as the percentage of sites affected per participant. Plaque and calculus will be assessed using standard indices, and tooth mobility will be graded using the Miller classification. The number of missing teeth (excluding third molars) will be recorded, and the reason for each lost tooth will be recorded from participant reports and corroborated with clinical and radiographic findings where possible, categorized as caries, periodontitis, trauma, other, or unknown [29].

### Calibration of the Examiner

Before data collection, the examiner will undergo calibration by examining at least 10 patients who are not part of the study, twice within a 24-hour interval, to assess intraexaminer reliability for probing depth and clinical attachment level. Intraclass correlation coefficients will be calculated, and a value of 0.80 or higher will be considered acceptable. Recalibration will be performed periodically as needed. The examiner will also be trained in the standardized aMMP-8 testing protocol.

### aMMP-8 PoCT

Participants will be asked to abstain from food, fluids, chewing gum, and toothbrushing for at least 1 hour before sample collection, and samples will be collected between 10 AM and noon to minimize diurnal variation. After a prerinse with tap water, participants will perform a 30-second rinse with 5 mL of purified water supplied in the test kit, and the rinse will be collected in the provided cup. Qualitative analysis will be performed immediately using a commercial lateral-flow mouth-rinse immunoassay (PerioSafe, Dentognostics GmbH, or equivalent) with a 20 ng/mL cutoff, and results will be recorded as positive (20 ng/mL or higher) or negative (below 20 ng/mL) according to the manufacturer’s instructions. For quality assurance, a subset of samples (approximately 10%) will be stored at −20 °C for potential quantitative enzyme-linked immunosorbent assay validation.

### Statistical Analysis

Analyses will be conducted in SPSS (version 27; IBM Corp), with 2-sided tests and a significance threshold of *P*<.05. Continuous variables (eg, age, T-scores, mean probing depth, and clinical attachment loss) will be summarized as means and SDs, or medians and IQRs when not normally distributed; categorical variables (eg, BMD category, smoking status, aMMP-8 positivity, and periodontitis stage) will be summarized as frequencies and percentages. Between-group comparisons will use independent *t* tests or Mann-Whitney *U* tests for continuous variables and chi-square or Fisher exact tests for categorical variables. The prevalence of periodontitis (overall and by stage and grade) and of tooth loss will be estimated in each group with 95% CIs.

For the primary analysis, the primary end point (periodontitis present vs absent) will be compared between the low-BMD and normal-BMD groups using the McNemar test applied to the 1:1 age-matched pairs, with the matched odds ratio and 95% CI estimated by conditional logistic regression; a multivariable conditional logistic regression model will additionally adjust for smoking status, systemic conditions, and education. Periodontitis severity (ordinal AAP and EFP stage) will be compared across matched pairs using the Stuart-Maxwell test of marginal homogeneity and modeled with proportional-odds ordinal regression. The diagnostic performance of aMMP-8 PoCT (sensitivity, specificity, and positive and negative predictive values) will be calculated using the clinical periodontal diagnosis as the reference standard, with receiver operating characteristic analysis if quantitative aMMP-8 data are available. Correlations between aMMP-8 levels and periodontal parameters and between BMD T-scores and periodontal indicators will be assessed using correlation analysis and regression modeling. Multivariable logistic regression will identify predictors of periodontitis, adjusting for age, smoking, systemic conditions, and BMD status; linear regression will be used for continuous outcomes such as mean clinical attachment loss; and ordinal or Poisson regression will be used for severity grades or tooth counts as appropriate. Nationality and education level will be included as covariates to control for demographic and socioeconomic variability. For continuous outcomes measured on matched pairs (eg, mean probing depth and clinical attachment loss), paired *t* tests or Wilcoxon signed-rank tests will be used, and McNemar or marginal homogeneity tests will be used for other paired categorical outcomes, so that the 1:1 matched design is retained throughout the primary comparisons [30]. Final analyses will be reviewed with a qualified biostatistician.

### Ethical Considerations

The study has received approval from the HMC Institutional Review Board (MRC-01-25-782; initial approval September 1, 2025) and will be conducted in accordance with the Declaration of Helsinki. Potential participants will be identified from records of women aged 50 years or older attending the geriatric clinics who have undergone DXA scanning. They will be screened for eligibility with appropriate data-access approvals and approached during a clinic visit or by telephone. Age-matched controls will be recruited from the same clinic population using the same process. Eligible women will receive a detailed explanation of the study’s purpose, procedures, duration, risks, benefits, and confidentiality safeguards in a private setting and will be given adequate time to consider participation and ask questions. Written informed consent, on an institutional review board (IRB)–approved form available in Arabic and English, will be obtained before any study procedure, and participants will receive a copy of the signed consent form. Participants may withdraw at any time; for withdrawn participants, no further data will be collected, and data already collected may be used in the analysis.

The study involves predominantly noninvasive procedures (oral examination, oral rinse collection, and review of existing DXA scans), which carry minimal risk. The periodontal examination may cause slight discomfort or minor transient bleeding, and the aMMP-8 oral rinse procedure is noninvasive. Serious adverse events are not anticipated; any adverse or unanticipated events will be assessed by the research team and reported through the hospital incident-reporting system and to the IRB.

## Results

This protocol describes a cross-sectional study that is in its data collection phase; no outcome data are available at the time of submission. The study received ethics approval on September 1, 2025 (MRC-01-25-782), and it is funded by the HMC Medical Research Center.

The current status of the study is as follows. Funding was awarded in January 2026. Screening of eligible participants who met the inclusion criteria started in June 2026. Recruitment is anticipated to commence in October 2026 and is expected to be completed by October 2027. The primary results are anticipated for publication in early 2028.

Once data collection is complete, the study will report the prevalence of periodontitis (overall and by stage and grade) and of tooth loss in older women with low BMD and in age-matched controls, together with between-group comparisons. Planned secondary outputs include mean clinical periodontal parameters (probing depth, clinical attachment loss, and percentage of sites with bleeding on probing) in each group; the mean number of missing teeth and the distribution of reasons for tooth loss; the prevalence of aMMP-8 positivity in oral rinse and its diagnostic performance for periodontitis; correlations between aMMP-8 levels and clinical periodontal parameters; the association between BMD status and aMMP-8 positivity; and the demographic, medical, geriatric, and lifestyle factors associated with periodontitis, tooth loss, and aMMP-8 positivity. The reporting of results will follow the STROBE recommendations for cross-sectional studies [21].

## Discussion

### Anticipated Principal Findings

This study is designed to test the hypothesis that older women with low BMD have a higher prevalence and greater severity of periodontitis, as well as increased tooth loss, than age-matched women with normal BMD. On the basis of prior work, we anticipate that the low-BMD group will show a higher periodontitis prevalence and higher mean clinical attachment loss, probing depth, and bleeding on probing, and that aMMP-8 positivity in oral rinse will be more frequent in this group and will correlate with clinical periodontal parameters. We further anticipate that aMMP-8 PoCT will demonstrate moderate diagnostic performance against the clinical periodontal diagnosis, supporting its potential role as a screening adjunct. Because the design is cross-sectional, any observed relationships will describe associations at a single time point and will not establish causal or temporal direction.

### Comparison With Prior Work

The anticipated direction of the primary finding is consistent with a systematic review and meta-analysis reporting greater clinical attachment loss, probing depth, gingival recession, and bleeding on probing in postmenopausal women with osteoporosis than in those without [9], and with observational data linking low BMD to periodontal attachment loss in perimenopausal and postmenopausal women [5,7]. Reviews of the periodontitis-osteoporosis relationship describe shared risk factors, including age, smoking, and hormonal change, and shared inflammatory mechanisms in which proinflammatory cytokines such as interleukin-1, interleukin-6, and tumor necrosis factor-α promote osteoclast-mediated bone resorption, which may reduce both systemic and alveolar bone density and increase susceptibility to periodontal breakdown [5,6]. By combining validated periodontal assessment with aMMP-8 PoCT in a Middle Eastern geriatric population, the study will extend this literature, in which chairside aMMP-8 testing has been evaluated mainly in other settings and populations [11-13].

### Strengths and Limitations

Strengths of the study include the use of a single calibrated examiner with predefined reliability thresholds, the application of the contemporary 2017 classification, individual age-matching of cases and controls, and the integration of a biomarker (aMMP-8) with comprehensive geriatric assessment in a population that is comparatively understudied locally. Several limitations should also be acknowledged. The cross-sectional design permits assessment of associations only and cannot establish causality or temporal sequence. Recruitment from a single institution may limit generalizability to all older women in Qatar and elsewhere, and the age-matched control group may differ in unmeasured factors. Self-reported information, such as the reason for tooth loss and elements of medical history, is subject to recall bias. Although calibration will be performed, some measurement variability is inherent in clinical periodontal assessment. The aMMP-8 PoCT provides qualitative results; diurnal variation may persist despite standardized timing; and testing is limited to oral rinse. Participant adherence to precollection instructions may also vary. Finally, because the study does not include an intervention, direct participant benefit is limited to assessment and appropriate referral.

### Future Directions

Although cross-sectional, the study is intended to provide a foundation for future longitudinal research, including studies of the temporal relationship between low BMD and periodontal disease and of the effect of periodontal treatment on bone and geriatric health outcomes in the local population. If aMMP-8 PoCT demonstrates adequate performance, subsequent work could evaluate its implementation as a screening adjunct within integrated geriatric-dental pathways.

### Dissemination

The findings will be disseminated through publication in peer-reviewed journals in periodontology, geriatrics, or bone health and through presentation at relevant local and international scientific meetings and internal academic events at HMC. Authorship will follow the International Committee of Medical Journal Editors criteria. Beyond publication, the results may inform the development of Qatar-specific, multidisciplinary screening protocols that integrate dental, bone health, and geriatric assessment and may support future interventional research and care coordination models within HMC and national geriatric services.

### Conclusions

This protocol describes a cross-sectional study that will characterize the prevalence and severity of periodontitis and tooth loss among older women with low BMD compared with age-matched controls and will evaluate aMMP-8 oral rinse PoCT as a supporting screening tool. The study will provide local evidence on the relationship between skeletal and oral health in older women and will inform integrated preventive and referral strategies. Consistent with its observational design, the study will describe associations rather than establish causation, and its conclusions will be framed accordingly.

## Supplementary material

10.2196/96991 Checklist 1 STROBE checklist.

10.2196/96991 Peer Review Report 1 Peer review report by Hamad Medical Corporation, Medical Research Centre (Qatar).

## Abbreviations

AAP: American Academy of Periodontology
aMMP-8: active matrix metalloproteinase-8
BMD: bone mineral density
DXA: dual-energy X-ray absorptiometry
EFP: European Federation of Periodontology
HMC: Hamad Medical Corporation
IRB: institutional review board
PoCT: point-of-care testing
SPIRIT: Standard Protocol Items: Recommendations for Interventional Trials
STROBE: Strengthening the Reporting of Observational Studies in Epidemiology
