# Supplementary material for: Prevalence of Periodontitis and Tooth Loss Among Older Adult Women With Low Bone Mineral Density: Protocol for a Cross-Sectional Study
Source: JMIR Res Protoc. 2026 Aug 28;15:e96991. doi: 10.2196/96991 (PMC13524217; doi:10.2196/96991)
Supplement: Supplementary file 1 [file resprot-v15-e96991-s002.pdf]

Date: 16/07/2025
Proposal Number: MRC-01-25-782
Proposal Title: Prevalence of Periodontitis and Tooth Loss Among older adult females with Low Bone Mineral Density

SUBJECT: Scientific Review Response Letter

Kindly adhere to the listed instructions for an efficient review process:

- Please respond **objectively in a point-by-point manner** to the reviewer's comments, **indicating where necessary the page number** where revisions have been made.
- Please upload the revised documents along with the response letter. To avoid duplication, please ensure that you are replacing the previous document and not uploading a new document.
- Kindly note that revisions in any document must be highlighted in a different color, such as **Purple for Reviewer A**
- Please include your responses to the reviewer's comments **in this document only** and **NOT** through ABHATH clarifications. Responses received through clarifications will not be considered.

REVIEWER'S COMMENTS:

1) Originality:

Reviewer A: I don't think such a study is done in Qatari population, so it's definitely novel

Response: Thank you for recognizing the novelty of our study. To the best of our knowledge, this is the first study in Qatar to investigate the association between low bone mineral density and periodontitis among elderly women, incorporating both clinical and biochemical diagnostic parameters (including aMMP-8 point-of-care testing). We believe that the local context and integration of multidisciplinary screening approaches contribute significantly to the originality and relevance of this research within the Qatari population and the broader Middle Eastern region.

2)Experimental Design & Analytical Methodology:

Reviewer A: The proposal is detailed; however, certain areas require improvement, particularly regarding the specification of the primary outcome and the threshold definition for periodontitis. Specific comments addressing these issues are provided below.

Response:

Thank you for your constructive feedback. We have clarified the primary outcome as the prevalence of periodontitis (categorized by stage/grade using the 2017 AAP/EFP classification system) among elderly women with low bone mineral density, compared to age-matched controls with normal BMD.

Additionally, the threshold **definition for periodontitis** has now been explicitly stated based on standard diagnostic criteria using full-mouth clinical periodontal parameters probing depth (PD), clinical attachment level (CAL), and bleeding on probing (BOP)with severity staged according to AAP/EFP guidelines. These updates are reflected in the revised sections on Study Objectives and Study Procedures.

We appreciate your suggestion and believe these clarifications enhance the scientific rigor and clarity of our protocol.

3) Feasibility:

Reviewer A: The study appears feasible, although details regarding patient flow and the capabilities of the research team remain unclear. As this is primarily an observational study rather than an intervention-based one, feasibility is likely. However, it should be clarified whether treatment will be provided to the participating patients.

Response:

Thank you for your thoughtful comments on feasibility. We agree that this study is observational in nature, which supports its overall practicality. We have now included further clarification in the revised protocol regarding:

1. Patient Flow:

Eligible participants will be identified from the existing appointment schedules and DXA scan records at the Geriatric Clinic at Rumailah Hospital. Screening and recruitment will take place during regular clinic visits, ensuring minimal disruption to clinical workflow. Oral examinations and aMMP-8 sample collection will be scheduled within the same visit whenever possible.

2. Research Team Capabilities:
   The clinical assessments will be conducted by a single, calibrated examiner with extensive experience in periodontal diagnostics. The co-investigators from HMC and UDST bring substantial clinical and academic expertise in geriatric medicine, dental hygiene, and epidemiological research. The study team also includes trained research staff to manage data collection, consent, and logistics.
3. Treatment for Participants:
   As this is a non-interventional study, no direct treatment will be provided as part of the research protocol. However, participants found to have clinical signs of moderate or severe periodontitis will be referred to the appropriate dental services within HMC for further evaluation and management, as per standard care procedures.

4) Importance to Qatar & Impact:

Reviewer A: Since the study is novel and focuses on postmenopausal women, it has the potential for a significant impact. It could also serve as a foundation for future longitudinal studies in which treatment is provided, thereby enhancing the overall value of the research.

Response:

Thank you for recognizing the significance and potential impact of our study. We agree that the focus on postmenopausal women fills a critical data gap within the Qatari healthcare context, especially as the country's population ages.

The study is designed not only to provide baseline epidemiological data on the association between periodontitis and low bone mineral density in elderly women, but also to inform Qatar-specific clinical guidelines, improve multidisciplinary screening practices, and support policy planning aligned with Qatar's National Health Strategy and Vision 2030.

We also appreciate your suggestion regarding future longitudinal work. Indeed, this cross-sectional study aims to serve as a launching platform for subsequent longitudinal or interventional studies, particularly those evaluating the effect of targeted periodontal treatment on bone health outcomes. These future studies would enhance evidence-based integration of dental and geriatric care across Qatar.

Revisions reflecting this long-term vision and relevance have been added to the Introduction, Study Rationale, and Dissemination of Results and Publication Policy sections.

5) Data Management & Statistical methods:

Reviewer A: I am not an expert in statistics, broadly, most of the areas seem to have been covered.

Response:

Thank you for your comment. We appreciate your acknowledgement that the data management and statistical analysis plan is comprehensive. To further ensure transparency and reproducibility, we have reviewed and retained all key components, including:

- Clear definitions of outcome variables and covariates
- Prevalence comparisons using Chi-square and t-tests as appropriate
- Multivariable logistic and linear regression models to control for confounding
- Diagnostic accuracy assessment of aMMP-8 PoCT using sensitivity, specificity, and predictive values
- Use of correlation analysis and ROC curves, where applicable

We are confident that the current statistical plan (Section 9) aligns with the objectives and design of this cross-sectional study. We remain open to consultation with a biostatistician for final analysis validation before publication.

6) Further comments

Reviewer A: It's a well written research proposal evaluating the prevalence of periodontitis and tooth loss in elderly women with low BMD and compare it to the prevalence in age-matched controls with normal BMD, according to the 2018 AAP/EFP classification system

The research proposal can be improved with the following additions:

1. The term resident needs to be clearly defined. Does it refer specifically to Qatari citizens, or does it include all individuals residing in Qatar, regardless of nationality? If it includes non-citizen residents, such as individuals from the Asian subcontinent, potential sources of bias may arise due to differences in socioeconomic status, genetic background, and other demographic factors. Taking just Qatari citizens, would mean difficulty to reach the sample size?? Please decide on this based on the logistics.

Thank you for your thoughtful observation. We agree that the term "resident" should be clarified to avoid ambiguity and ensure transparent reporting.

In the revised protocol, we have specified that "resident" refers to all women aged 50 and older residing in Qatar who attend the Geriatric Clinics at Hamad Medical Corporation, regardless of nationality. This approach reflects the demographic

diversity of the patient population served by HMC and enhances feasibility for achieving the required sample size.

We recognize that including non-citizen residents may introduce variability related to genetics, socioeconomic status, and lifestyle, which we will address during data analysis by collecting detailed demographic data (e.g., nationality, education level, income proxy measures) and incorporating these variables as potential confounders in multivariable regression models.

This inclusive definition also supports broader generalizability of the findings across Qatar's aging population, which includes a substantial proportion of long-term expatriates.

We have updated the relevant sections in the Study Population, Inclusion Criteria, and Statistical Analysis Plan to reflect this clarification.

2. My main concern is regarding the primary outcome. Usually, in any study, the primary outcome should be only one and not multiple primary outcomes. The primary outcome needs to be more specific. The term prevalence of periodontitis is too broad.
   Suggestion: It is recommended to define the primary outcome more precisely, such as the prevalence of periodontitis based on clinical parameters like probing depth (PD) ≥ 5 mm, clinical attachment loss (CAL) ≥ 5 mm, or periodontal inflamed surface area (PISA). This is a mandatory correction.

Thank you for your important observation regarding the primary outcome. We agree that greater clarity and specificity are needed. We would like to clarify that the primary outcome of our study is the presence of periodontitis and its distribution across different severity grades, as classified by the 2017 AAP/EFP Classification of Periodontal and Peri-Implant Diseases and Conditions. This classification is based on clinical attachment loss (CAL), probing depth (PD), and radiographic bone loss, and allows for categorization into stages (I–IV) and grades (A–C), rather than relying solely on arbitrary thresholds like PD ≥5 mm or CAL ≥5 mm.
Our objective is to determine whether a relationship exists between low bone mineral density (BMD) and periodontitis, and if so, to evaluate how the severity of periodontitis correlates with the degree of BMD reduction (osteopenia vs. osteoporosis).
To meet your suggestion and ensure methodological rigor, we have:

- Clearly defined periodontitis staging criteria according to AAP/EFP 2017 in the protocol
- Designated a single primary outcome as:

"Prevalence of periodontitis (as classified by AAP/EFP 2017 staging system) among elderly women with low BMD compared to controls."

- Moved tooth loss and other periodontal metrics (mean PD, CAL, BOP%, aMMP-8 levels) to secondary outcomes

These clarifications have been reflected in the Objectives, Study Outcomes, and Statistical Analysis sections of the revised protocol.

3. Another suggestion for a primary outcome could be PISA. PISA is a composite index combining pocket depth with bleeding on probing. Below is the article for using PISA as a primary outcome. Nesse W, Abbas F, van der Ploeg I, Spijkervet FK, Dijkstra PU, Vissink A. Periodontal inflamed surface area: quantifying inflammatory burden. J Clin Periodontol. 2008 Aug;35(8):668-73. The forms to calculate the PISA are given in this link if the grant allows hiring a research assistant, the PI can relegate the calculation.

Thank you for this insightful suggestion. We fully acknowledge the clinical and research value of the **Periodontal Inflamed Surface Area (PISA)** as an advanced composite index for assessing the inflammatory burden in periodontal tissues. The referenced study by Nesse et al. (2008) is indeed foundational in highlighting PISA's role in quantifying systemic inflammatory load.

However, for the purposes of this study, we have chosen to adopt the **2017 AAP/EFP classification system** for diagnosing and staging periodontitis, as it represents the **most widely accepted, internationally standardized method** for clinical and epidemiological research. This system allows categorization based on a combination of clinical attachment loss (CAL), probing depth (PD), and radiographic bone loss, and supports consistent classification into stages (I–IV) and grades (A–C).

Moreover, as our primary aim is to assess the **association between bone mineral density (BMD) and both the presence and severity of periodontitis**, using a recognized diagnostic framework is essential to maintain comparability with other global studies and to support potential clinical translation.

While PISA provides valuable adjunct information, it is not yet universally established as a diagnostic or surveillance criterion for periodontitis, and therefore is **not suitable as the primary outcome** in this context.

Should resources permit, we are open to including PISA as an **exploratory secondary outcome** in future research to further quantify inflammatory burden and its link to systemic conditions.

This rationale has been clarified in the revised **Study Objectives** and **Study Outcomes** sections of the protocol.

4. Another key concern which needs to be addressed is the definition of periodontitis

Merely stating periodontitis will be diagnosed based on 2018 classification is not sufficient. For example, there is 50-year-old post-menopausal women, with two sites with interproximal CAL of 1-2 mm is considered as stage I periodontitis.
In another context, there is 50-year-old post-menopausal women, with 25 sites with interproximal CAL of ≥ 5 mm is considered as stage III/IV, Grade C periodontitis
This is regarding the severity of periodontitis. Other aspect is regarding localised or generalised. According to 2018 classification, 2 sites with interproximal CAL is also considered as periodontitis, and 30 sites with interproximal CAL is also considered as periodontitis. How will the stats guy handle this data. If out of 200 controls, half are localised and half are generalised periodontitis

This clarification is crucial since saliva samples are being collected to measure MMP-8 levels. Notably, salivary MMP-8 levels differ significantly between generalized and localized periodontitis. Given that periodontal disease is site-specific, measuring MMP-8 from gingival crevicular fluid (GCF) would reflect the biomarker level at the specific diseased site.

Suggestion: While using the 2018 classification for periodontitis is appropriate, the study should clearly define a diagnostic threshold. For instance, periodontitis could be defined as the presence of ≥6 sites with interproximal pocket depths >5 mm. Alternatively, the study could include only participants with generalized periodontitis to ensure a more homogenous sample and reliable interpretation of salivary MMP-8 levels.

Response:
Thank you for bringing this important and clinically relevant concern to our attention. We agree that **clear operationalization of the definition and severity of periodontitis is essential** for consistency in data interpretation and biomarker correlation. We confirm that we are using the **2017 AAP/EFP classification system** (not the 2018 system), which provides a robust diagnostic and staging framework for periodontitis. In response to your recommendation, we have now added the following **diagnostic thresholds and definitions** to the protocol for consistency in classification and analytical interpretation:

Definition in Study Protocol (To Be Added in Methods & Outcomes)
Diagnosis of Periodontitis (As Per 2017 AAP/EFP Criteria):

Case definition for periodontitis: ≥ **2 non-adjacent interproximal sites** with **clinical attachment loss (CAL) ≥3 mm**, AND ≥ **1 site** with **probing depth (PD) ≥4 mm** OR CAL ≥3 mm and PD ≥5 mm on the same site.

Staging Criteria:
- **Stage I:** CAL 1–2 mm
- **Stage II:** CAL 3–4 mm
- **Stage III/IV:** CAL ≥5 mm at multiple sites with possible tooth loss, vertical bone loss, or mobility

Extent Definitions:

- **Localized:** ≤30% of teeth involved
- **Generalized:** >30% of teeth involved

We will record both **stage (I–IV)** and **extent (localized vs. generalized)** for every participant and use these as stratification variables in the statistical analysis.

MMP-8 Implications:

We appreciate your insight regarding the **variation of salivary aMMP-8 levels between localized and generalized periodontitis**. Although aMMP-8 is not site-specific like GCF-based analysis, salivary MMP-8 has shown clinical utility in **identifying disease presence and severity level**, which aligns with our objective of examining systemic oral inflammation in the context of BMD.

To account for potential heterogeneity in MMP-8 readings, we will:

- Analyze aMMP-8 levels **stratified by generalized vs. localized disease**
- Include **extent and stage** of periodontitis as independent variables in multivariable regression models

5. An additional suggestion to strengthen the study would be to include an assessment of local bone density. If funding permits, consider obtaining CBCT scans for a subset of participants—this does not need to include the entire sample, but could focus on a representative subgroup, particularly those with missing teeth in the maxilla or mandible. From the CBCT images, Hounsfield Unit (HU) values can be measured at three distinct points within the region of interest, and the average HU value can then be calculated. This would enable the study to integrate clinical periodontal parameters, MMP-8 biomarker data, systemic BMD, and localized bone density, providing a more comprehensive understanding of the relationship between systemic and local bone conditions. This would help in publishing in a high impact Q1 journal or may will give one Q1 and another Q2 article.

   Thank you for this excellent and forward-thinking suggestion. We fully agree that incorporating **CBCT-based local bone density assessment** would significantly enrich the study by allowing correlation between **localized skeletal structure and systemic BMD**, alongside periodontal and biomarker data.

   However, at this stage, due to current **resource and logistical limitations**, we are unable to diversify the present study protocol to include CBCT imaging. Nevertheless, we **recognize the scientific merit** of this addition and will **strongly consider including CBCT scans in a subset of participants as part of a future or follow-up study**. This could provide valuable data for more in-depth analysis and support a focused publication on localized bone–periodontal relationships.

6. O' leary plaque score needs to be scored.
   O'Leary TJ, Drake RB, Naylor JE. The plaque control record. J Periodontol. 1972;43(1):38.

   Thank you for your valuable suggestion. We agree that incorporating the **O'Leary Plaque Control Record** will enhance the thoroughness of the clinical periodontal assessment by providing a standardized measure of oral hygiene status.

7. Research consent form is good

   Thank you for your positive feedback. We appreciate your acknowledgement of the research consent form. It has been designed to ensure clarity, transparency, and compliance with **ethical and regulatory standards**, including Good Clinical Practice (GCP) and the Institutional Review Board (IRB) requirements at HMC. We will continue to ensure that participants are fully informed and that their autonomy is respected throughout the study.

8. Convert the questionnaire into an online format using Google Forms, SurveyMonkey, or Qualtrics. For elderly patients or those who are not tech-savvy, the questionnaire should be administered by a trained staff member rather than simply forwarded for self-completion, as all key variables are captured through this instrument. Prior to full implementation, pilot the questionnaire on a small sample of patients to ensure clarity and reliability—similar to the planned calibration process for clinical examination.

   Thank you for this helpful and practical recommendation. We recognize the value of digitizing the questionnaire to improve data quality and streamline collection. We will strongly consider converting the tool into an online format (e.g., Google Forms or Qualtrics) during the implementation phase, based on guidance from the Institutional Review Board (IRB) and operational feasibility. In alignment with your suggestion, we also agree that the questionnaire should be administered by trained research staff for elderly participants or those with limited digital literacy, and that pilot testing will be essential to assess clarity and reliability.
   These considerations will be incorporated into our operational planning, subject tIRB approval and resource availability.
