# Supplementary material for: Prevalence of Periodontitis and Tooth Loss Among Older Adult Women With Low Bone Mineral Density: Protocol for a Cross-Sectional Study
Source: JMIR Res Protoc. 2026 Aug 28;15:e96991. doi: 10.2196/96991 (PMC13524217; doi:10.2196/96991)
Supplement: Supplementary file 2 [file resprot-v15-e96991-s001.docx]

**STROBE Statement Checklist**

**Manuscript Title:** *Prevalence of Periodontitis and Tooth Loss Among Older Adult Women With Low Bone Mineral Density: Protocol for a Cross-Sectional Study*

*Note: Because this manuscript is a study protocol, items in the Results section (Items 13–17) reflect planned data collection and methodological reporting rather than completed empirical outcome data.*

| **Item No.** | **Item / Topic** | **Location** | **Relevant Text & Details** |
| --- | --- | --- | --- |
| **TITLE & ABSTRACT** | | | |
| **1 (a)** | Title / Abstract Design | Title / Abstract | Indicate study’s design with a commonly used term in the title or abstract.  • Title: "...Protocol for a Cross-Sectional Study" • Abstract (Methods): "This single-center, cross-sectional study will recruit postmenopausal women..." |
| **1 (b)** | Abstract Summary | Abstract | Provide an informative and balanced summary of what was done and what was found.  • Covers Background, Objective, Methods, Results (current protocol status/timeline), and Conclusions. |
| **INTRODUCTION** | | | |
| **2** | Background / Rationale | Introduction (p. 1–2) | Explain scientific background and rationale for the investigation.  • "Periodontitis and low bone mineral density (BMD) are both highly prevalent among older women and share inflammatory and metabolic pathways..." |
| **3** | Objectives | Objectives (p. 2) | State specific objectives, including prespecified hypotheses.  • "The primary objective is to determine and compare the prevalence and severity of periodontitis... among older women with low BMD... and age-matched women with normal BMD." |
| **METHODS** | | | |
| **4** | Study Design | Methods: Study Design (p. 2) | Present key elements of study design early in the paper.  • "This study will use a cross-sectional design to investigate the prevalence of and associations among periodontitis, tooth loss, aMMP-8 levels in oral rinse, and low BMD in women aged 50 years or older." |
| **5** | Setting | Methods: Study Setting / Results (p. 2–4) | Describe setting, locations, relevant dates, recruitment, and data collection.  • Setting: "...geriatric clinics of Rumailah Hospital, Hamad Medical Corporation, Doha, Qatar." • Dates: Screening started June 2026; recruitment planned Oct 2026 – Oct 2027. |
| **6 (a)** | Participants (Selection) | Methods: Study Setting & Participants / Textbox 1 (p. 2–3) | Eligibility criteria, sources, and methods of selection.  • Postmenopausal women aged ≥50 years attending geriatric clinics. Full inclusion/exclusion listed in Textbox 1. |
| **6 (b)** | Participants (Matching) | Methods: Matching of Cases & Controls (p. 3) | Matching criteria and numbers (if applicable).  • Controls individually matched 1:1 to cases by age within 3 years. |
| **7** | Variables | Objectives / Methods: Periodontal Exam & aMMP-8 (p. 2–4) | Clearly define outcomes, exposures, predictors, potential confounders, and effect modifiers.  • Exposure: Low BMD (T-score ≤ -1.0) vs Normal BMD (T-score > -1.0). • Primary Outcome: Periodontitis presence/stage (2017 AAP/EFP criteria). • Secondary: aMMP-8 positivity, tooth loss, BOP, PD, CAL. • Confounders: Age, smoking status, education, comorbidities. |
| **8** | Data Sources / Measurement | Methods: Data Collection / Periodontal Exam / aMMP-8 (p. 3–4) | Sources of data and details of assessment methods.  • DXA reports from electronic records; full-mouth 6-site exam via UNC-15 probe by calibrated examiner; PerioSafe PoCT lateral-flow immunoassay (20 ng/mL cutoff). |
| **9** | Bias | Methods: Study Design / Matching / Calibration (p. 2–4) | Describe any efforts to address potential sources of bias.  • Single calibrated examiner (ICC ≥ 0.80); 1:1 age matching; standardized fasting and morning sample collection (10 AM–12 PM). |
| **10** | Study Size | Methods: Sample Size (p. 3) | Explain how the study size was arrived at.  • Sample calculation yields 170/group (340 total). Target set at 200/group (400 total) to allow ~18% non-response/data loss margin. |
| **11** | Quantitative Variables | Methods: Data Collection & Statistical Analysis (p. 3–4) | Explain how quantitative variables were handled in the analyses.  • BMD categorized via WHO criteria (Normal > -1.0, Osteopenia -1.0 to -2.5, Osteoporosis ≤ -2.5). Continuous variables summarized as mean (SD) or median (IQR). |
| **12 (a)** | Statistical Methods (Main) | Methods: Statistical Analysis (p. 4) | Describe statistical methods including confounder control.  • McNemar test and conditional logistic regression for primary matched pair comparison; multivariable conditional logistic regression adjusting for smoking, comorbidities, and education. |
| **12 (b)** | Subgroups & Interactions | Methods: Statistical Analysis (p. 4) | Describe methods used to examine subgroups and interactions.  • Proportional-odds ordinal regression for stage severity; ROC curves for aMMP-8 diagnostic performance. |
| **12 (c)** | Missing Data | Methods: Sample Size / Statistical Analysis (p. 3–4) | Explain how missing data were addressed.  • Over-recruitment of 400 total vs 340 minimum required to preserve complete matched pairs. |
| **12 (d)** | Matching / Sampling Strategy | Methods: Statistical Analysis (p. 4) | Describe analytical methods taking account of matching or sampling.  • Paired t-tests, Wilcoxon signed-rank, McNemar, and conditional logistic regression preserve 1:1 matched pair structure. |
| **12 (e)** | Sensitivity Analyses | Methods: Statistical Analysis / aMMP-8 PoCT (p. 4) | Describe any sensitivity analyses.  • Subgroup (~10%) of oral rinse samples frozen at -20°C for quantitative ELISA validation. |
| **RESULTS (Planned Protocol Reporting)** | | | |
| **13 (a)–(c)** | Participants | Abstract / Results (p. 1, 4) | Report numbers at each stage / flow diagram.  • ~600 screened to yield 400 enrolled (200 cases, 200 controls). |
| **14 (a)–(c)** | Descriptive Data | Methods: Statistical Analysis / Results (p. 4) | Characteristics of study participants and missing data.  • Planned demographic, clinical, and geriatric characterization tables. |
| **15** | Outcome Data | Methods / Results (p. 4) | Report outcome events or summary measures.  • Planned reporting of periodontitis prevalence, stages, mean PD/CAL, tooth loss counts, and aMMP-8 positivity. |
| **16 (a)–(c)** | Main Results | Methods: Statistical Analysis (p. 4) | Unadjusted/adjusted estimates, CIs, category boundaries.  • Planned reporting of matched Odds Ratios (OR) with 95% CIs via conditional logistic regression. |
| **17** | Other Analyses | Methods: Statistical Analysis (p. 4) | Report other analyses done (subgroups, interactions).  • Planned ROC curves for aMMP-8 and linear/ordinal regression models for systemic risk factors. |
| **DISCUSSION** | | | |
| **18** | Key Results | Discussion: Principal Findings (p. 5) | Summarise key results with reference to study objectives.  • Hypothesizes higher prevalence/severity of periodontitis and tooth loss in low-BMD group. |
| **19** | Limitations | Discussion: Strengths & Limitations (p. 5) | Discuss limitations, potential bias, or imprecision.  • Cross-sectional design (no causality); single-center setting; recall bias for tooth loss reasons. |
| **20** | Interpretation | Discussion: Principal Findings / Conclusions (p. 5–6) | Give cautious overall interpretation.  • Focuses on characterizing associations to inform integrated geriatric-dental screening pathways. |
| **21** | Generalisability | Discussion: Strengths & Limitations (p. 5) | Discuss generalisability (external validity).  • Clinical sample from Rumailah Hospital represents local geriatric population in Qatar. |
| **OTHER INFORMATION** | | | |
| **22** | Funding | Declarations: Funding (p. 6) | Source of funding and role of funders.  • Funded by Hamad Medical Corporation Medical Research Center (MRC-01-25-782). Funder had no role in study design or publication. |
